# Inhibitory effect of surface pre-reacted glass-ionomer (S-PRG) eluate against adhesion and colonization by *Streptococcus mutans*

**DOI:** 10.1038/s41598-018-23354-x

**Published:** 2018-03-22

**Authors:** Ryota Nomura, Yumiko Morita, Saaya Matayoshi, Kazuhiko Nakano

**Affiliations:** Department of Pediatric Dentistry, Division of Oral Infections and Disease Control, Osaka University Graduate School of Dentistry, Suita, Osaka Japan

## Abstract

Surface Pre-reacted Glass-ionomer (S-PRG) filler is a bioactive filler produced by PRG technology, which has been applied to various dental materials. A S-PRG filler can release multiple ions from a glass-ionomer phase formed in the filler. In the present study, detailed inhibitory effects induced by S-PRG eluate (prepared with S-PRG filler) against *Streptococcus mutans*, a major pathogen of dental caries, were investigated. S-PRG eluate effectively inhibited *S. mutans* growth especially in the bacterium before the logarithmic growth phase. Microarray analysis was performed to identify changes in *S. mutans* gene expression in the presence of the S-PRG eluate. The S-PRG eluate prominently downregulated operons related to *S. mutans* sugar metabolism, such as the *pdh* operon encoding the pyruvate dehydrogenase complex and the *glg* operon encoding a putative glycogen synthase. The S-PRG eluate inhibited several *in vitro* properties of *S. mutans* relative to the development of dental caries especially prior to active growth. These results suggest that the S-PRG eluate may effectively inhibit the bacterial growth of *S. mutans* following downregulation of operons involved in sugar metabolism resulting in attenuation of the cariogenicity of *S. mutans*, especially before the active growth phase.

## Introduction

*Streptococcus mutans* has been implicated as a primary causative agent of dental caries in humans^1^. Although the mechanisms of dental caries have been well investigated and the incidence of dental caries has been reduced in most developing countries, eradication of dental caries remains difficult^2^. Thus, novel dental products for prevention of dental caries are under development in various countries^3–5^.

Surface Pre-Reacted Glass-ionomer (S-PRG) fillers have been synthesized by PRG technology involving reaction between fluoroboroaluminosilicate glass and a polyacrylic acid solution^6^. The S-PRG filler is used in various dental materials including composite resins, bonding agents, cements, and resin sealants^7,8^. In addition, research focusing on the usefulness of S-PRG fillers in oral hygiene products such as mouthwashes to inhibit the bacteria or oral malodour has been reported^9^. S-PRG fillers can release six ions, fluoride (F^−^), sodium (Na^+^), borate (BO_3_^3−^), aluminium (Al^3+^), silicate (SiO_3_^2−^), and strontium (Sr^2+^), which have antimicrobial activity against various oral bacteria^10^. Although antimicrobial activity of S-PRG fillers against *S. mutans* has been reported^11–13^, the detailed inhibition mechanisms of S-PRG fillers remain unknown.

Dental caries development caused by *S. mutans* is induced by bacterial growth, survival and adhesion resulting in biofilm formation by microbial communities^14^. Sugar metabolism is an important factor for *S. mutans* growth and survival^15,16^, which is induced via the Embden-Meyerhof-Parnas pathway^16^. The sugar metabolic pathways including the Embden-Meyerhof-Parnas pathway are mainly observed in *S. mutans* during growth rather than in the stationary phase^17^.

In the present study, we investigated whether a S-PRG eluate prepared with a S-PRG filler can inhibit the bacterial growth of *S. mutans*. In addition, a molecular biological approach focusing on alterations in *S. mutans* gene expression in the presence of S-PRG eluate was performed using DNA microarray analysis. Furthermore, we analysed the inhibitory effects of the S-PRG eluate on several *in vitro* properties of *S. mutans* relavent to the development of dental caries.

## Results

### Inhibitory effects of S-PRG eluate on bacterial growth

**Figure 1:**
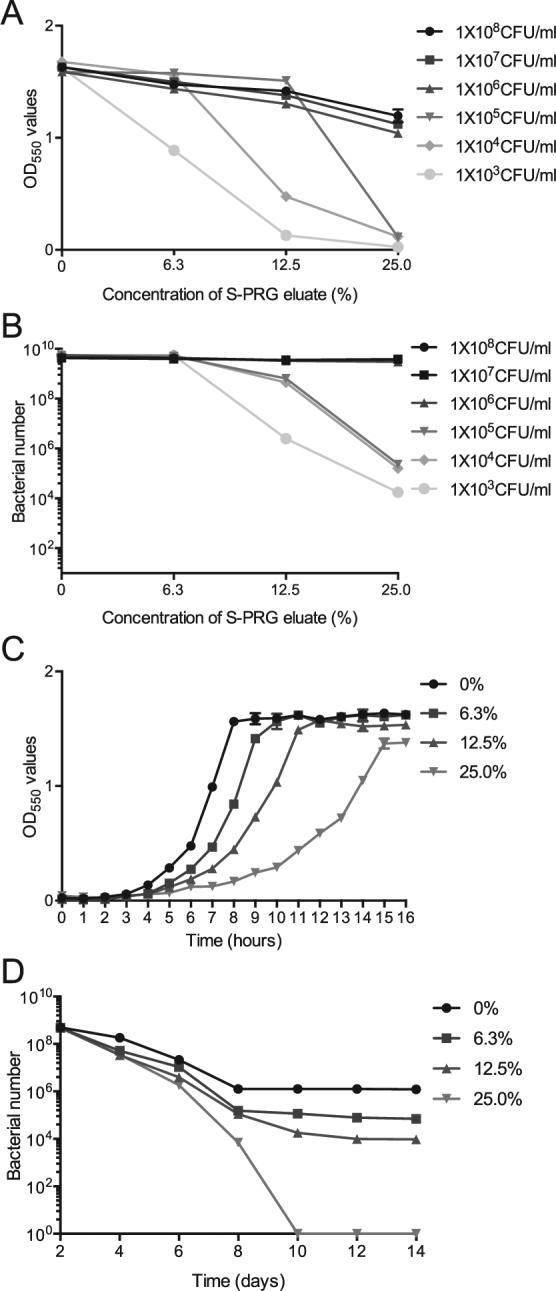
Inhibition on *S. mutans* MT8148 grown by the S-PRG eluate. (**A**,**B**) Bacterial growth by adding varying concentration of the eluate followed by 18 h incubation. Growth was determined by OD_550_ values in BHI broth (**A**) and recovered bacterial numbers on MSB plates (**B**). (**C**) Bacterial growth by adding 1 × 10^7^ CFU/ml of *S. mutans* at multiple time points, which was determined by OD_550_ values in BHI broth. (**D**) Bacterial survival by adding 1 × 10^7^ CFU/ml of *S. mutans* at multiple time points, which was determined by adding serial dilutions of the bacterial suspensions to MSB plates.

S-PRG eluate was added at final concentrations of 0%, 6.3%, 12.5% and 25.0% in brain heart infusion (BHI) broth (Difco Laboratories, Detroit, MI, USA). Bacterial suspensions were adjusted in the BHI broth with or without S-PRG eluate at final concentrations ranging from 1.0 × 10^3^ to 1.0 × 10^8^ CFU/ml. After 18-h incubation at 37 °C, bacterial growth was measured at OD_550_ and bacterial suspensions were then streaked onto Mitis Salivarius agar plates (Difco Laboratories) containing bacitracin (0.2 U/ml; Sigma-Aldrich, St. Louis, MO, USA) and 15% (w/v) sucrose (MSB agar), which were anaerobically cultured at 37 °C for 48 h. S-PRG eluate added to bacterial suspensions (1.0 × 10^3^ to 1.0 × 10^5^ CFU/ml in BHI broth) prominently inhibited bacterial growth, even after incubation at 37 °C for 18 h. This inhibition was S-PRG-concentration-dependent for both OD_550_ densities and bacterial numbers (Fig. 1A,B). Although slightly lower OD_550_ values were observed in bacterial suspensions with concentrations ranging from 1.0 × 10^6^ to 1.0 × 10^8^ CFU/ml, the test strains at concentrations >1.0 × 10^6^ CFU/ml did not show extensive reduction in cell numbers even when the S-PRG eluate was added at high concentrations. Thus, test strains adjusted to a final density of 1.0 × 10^7^ CFU/ml were not growth inhibited even in the presence of 25% of S-PRG eluate after 18-h incubation at 37 °C and were mainly used in subsequent studies. Next, we monitored the kinetics of growth inhibition of 1.0 × 10^7^ CFU/ml *S. mutans* in the presence of each concentration of S-PRG eluate before reaching the stationary phase. Bacterial growth of *S. mutans* without S-PRG eluate reached a plateau 7-h after incubation, and the lag times were lengthened in a dose dependent manner with the S-PRG eluate (Fig. 1C). The lag time for *S. mutans* to reach the stationary phase in the presence of 25% S-PRG eluate was approximately twice that in the absence of the S-PRG eluate. Furthermore, survival of 1.0 × 10^7^ CFU/ml *S. mutans* in the presence of each concentration of S-PRG eluate after the stationary phase was monitored (Fig. 1D). The recovered bacterial numbers were not different among *S. mutans* in the presence of each concentration of S-PRG eluate after two days of incubation. However, the numbers of recovered *S. mutans* were reduced in a dose dependent manner with the S-PRG eluate and no bacteria were recovered in the presence of 25% S-PRG eluate 10 days after incubation.

### DNA microarray assays

**Figure 2:**
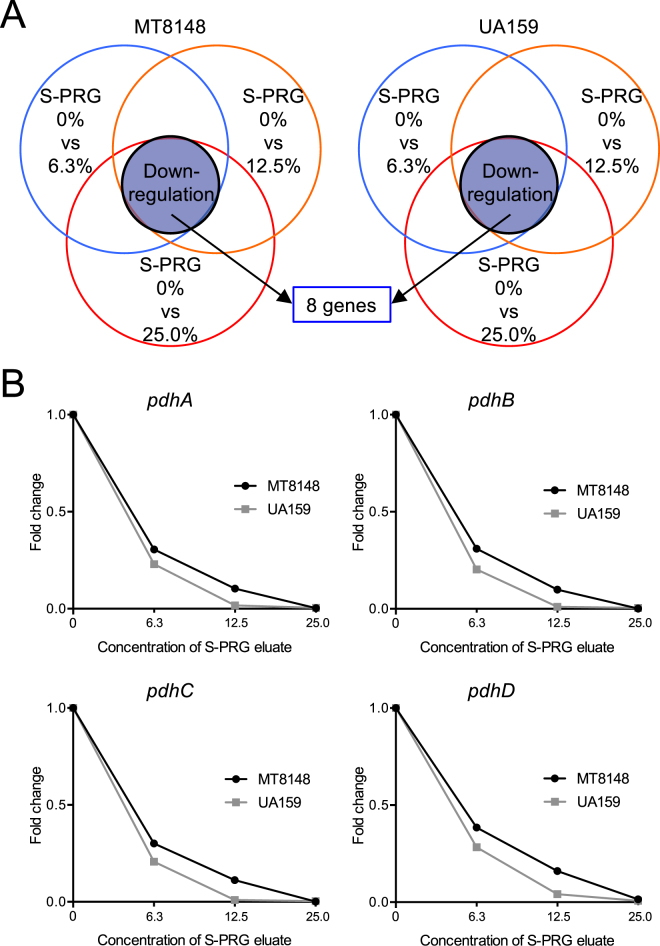
Altered expression of key genes in both *S. mutans* MT8148 and UA159 under three different conditions using DNA microarray analysis. (**A**) Detection of altered genes in the presence of S-PRG eluate. (**B**) Changes in the expression of genes in the *pdh* operon of MT8148 and UA159 in the presence of S-PRG eluate.

The S-PRG eluate could delay bacterial growth and inhibit bacterial survival of 1.0 × 10^7^ CFU/ml *S. mutans*; thus, we decided to identify key genes of *S. mutans* affected by the S-PRG eluate. 1.0 × 10^7^ CFU/ml of *S. mutans* MT8148 and UA159 in BHI broth were cultured with each specified concentration of S-PRG eluate at 37 °C for 18 h. Next, RNA samples were extracted from each sample for microarray analysis. In the microarray analysis, we evaluated three conditions at several concentrations of S-PRG eluate: 0% versus 6.3%, 0% versus 12.5%, and 0% versus 25.0%. From a list of genes, we selected those with increased or decreased changes of greater than 1.0 of Log2 ratio for comparison. First, we identified genes prominently regulated under all three conditions, which were identified in both *S. mutans* MT8148 and UA159, to reduce confounding effects of false signals (Fig. 2A). DNA microarray analysis revealed that eight genes were downregulated in all comparisons (Tables 1, 2). Among these genes, genes encoding the pyruvate dehydrogenase (PDH) complex, which plays an important role in *S. mutans* survival and is closely related to sugar metabolism^15,18^, were prominently downregulated. The PDH complex forms an operon containing four genes, *pdhD*, *pdhA*, *pdhB*, and *pdhC*^15^, all of which were downregulated by S-PRG eluate in a concentration-dependent manner in both MT8148 and UA159 (Fig. 2B).

**Table 1:** List of downregulated genes of MT8148 in the presence of S-PRG eluate, which were observed in both MT8148 and UA159 under three different conditions using DNA microarray analysis.

Gene	Accession number	S-PRG 0%	S-PRG 6.3%%			S-PRG12.5			S-PRG 25.0%		
		Signal	Signal	Log2*	Change#	Signal	Log2*	Change^#^	Signal	Log2*	Change#
*clp*	721354.1	10905.7	3934.7	−1.47	0.361	2604.3	−2.07	0.239	779.1	−3.81	0.071
*gldA*	720934.1	79738.3	15155.0	−2.40	0.190	4829.4	−4.04	0.061	2716.1	−4.88	0.034
*pdhA*	721781.1	157937.7	49355.6	−1.68	0.312	17366.4	−3.19	0.110	451.1	−8.45	0.003
*pdhB*	721780.1	118457.0	38021.4	−1.64	0.321	12313.0	−3.26	0.104	232.9	−9.05	0.002
*pdhC*	721779.1	147220.0	47212.9	−1.64	0.321	17231.7	−3.09	0.117	205.2	−9.49	0.001
*pdhD*	721782.1	20637.6	8184.3	−1.33	0.397	3493.2	−2.56	0.169	332.1	−5.98	0.016
*pfl2*	720932.1	16001.1	5582.2	−1.52	0.349	3688.0	−2.12	0.231	798.1	−4.33	0.050
*phsG*	721879.1	36063.9	13506.8	−1.42	0.375	6031.0	−2.58	0.167	1521.6	−4.57	0.042

^*^Log2 means Log2 ratio. ^#^Change means Fold change. The Log2 ratio and Fold change were calculated by signal in each concentration of S-PRG eluate/signal without S-PRG eluate.

**Table 2:** List of downregulated genes of UA159 in the presence of S-PRG eluate, which were observed in both MT8148 and UA159 under three different conditions using DNA microarray analysis.

Gene	Accession number	S-PRG 0%	S-PRG 6.3%			S-PRG12.5%			S-PRG 25.0%		
		Signal	Signal	Log2*	Change#	Signal	Log2*	Change^#^	Signal	Log2*	Change#
*clp*	721354.1	18865.6	6619.2	−1.51	0.351	2873.2	−2.71	0.152	3817.9	−2.30	0.202
*gldA*	720934.1	55312.5	21392.5	−1.37	0.387	9103.4	−2.60	0.165	2217.9	−4.64	0.040
*pdhA*	721781.1	30983.4	7458.1	−2.06	0.241	547.5	−5.82	0.018	151.5	−7.68	0.005
*pdhB*	721780.1	26341.2	5687.7	−2.21	0.216	283.9	−6.53	0.011	131.4	−7.69	0.005
*pdhC*	721779.1	32996.4	7188.0	−2.20	0.218	320.5	−6.69	0.010	128.0	−8.01	0.004
*pdhD*	721782.1	5817.6	1717.4	−1.76	0.295	250.6	−4.54	0.043	47.4	−6.95	0.008
*pfl2*	720932.1	11402.1	3593.0	−1.67	0.315	2135.1	−2.42	0.187	1288.9	−3.14	0.113
*phsG*	721879.1	41481.6	17147.4	−1.27	0.413	8112.9	−2.35	0.196	1380.1	−4.91	0.033

^*^Log2 means Log2 ratio. ^#^Change means Fold change. The Log2 ratio and Fold change were calculated by signal with each concentration of S-PRG eluate/signal without S-PRG eluate.

**Figure 3:**
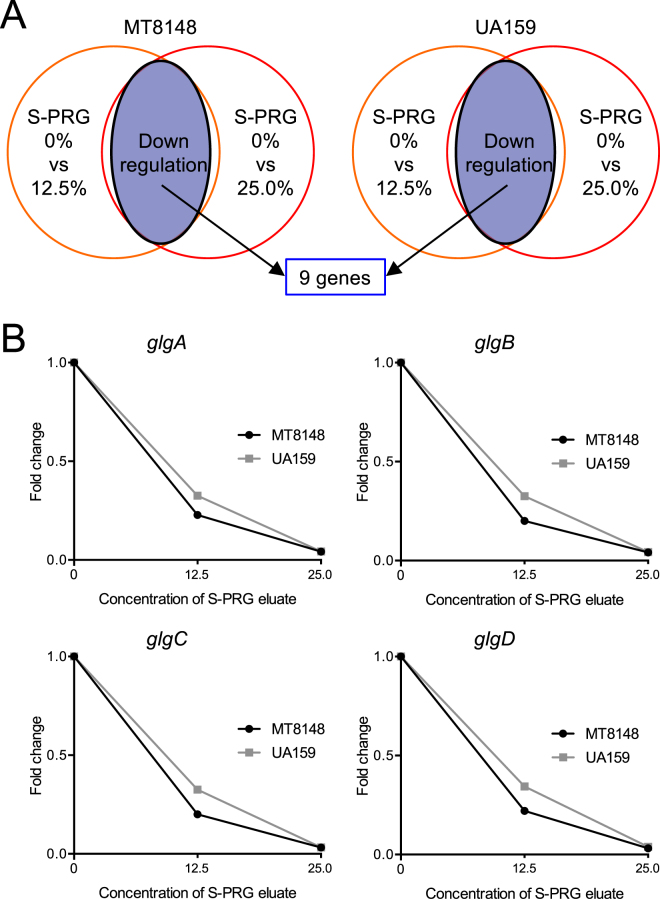
Altered expression of key genes in both *S. mutans* MT8148 and UA159 under two different conditions detected by DNA microarray analysis. (**A**) Detection of altered genes in the presence of S-PRG eluate. (**B**) Changes in the expression of genes in the *glg* operon of MT8148 and UA159 in the presence of S-PRG eluate.

**Figure 4:**
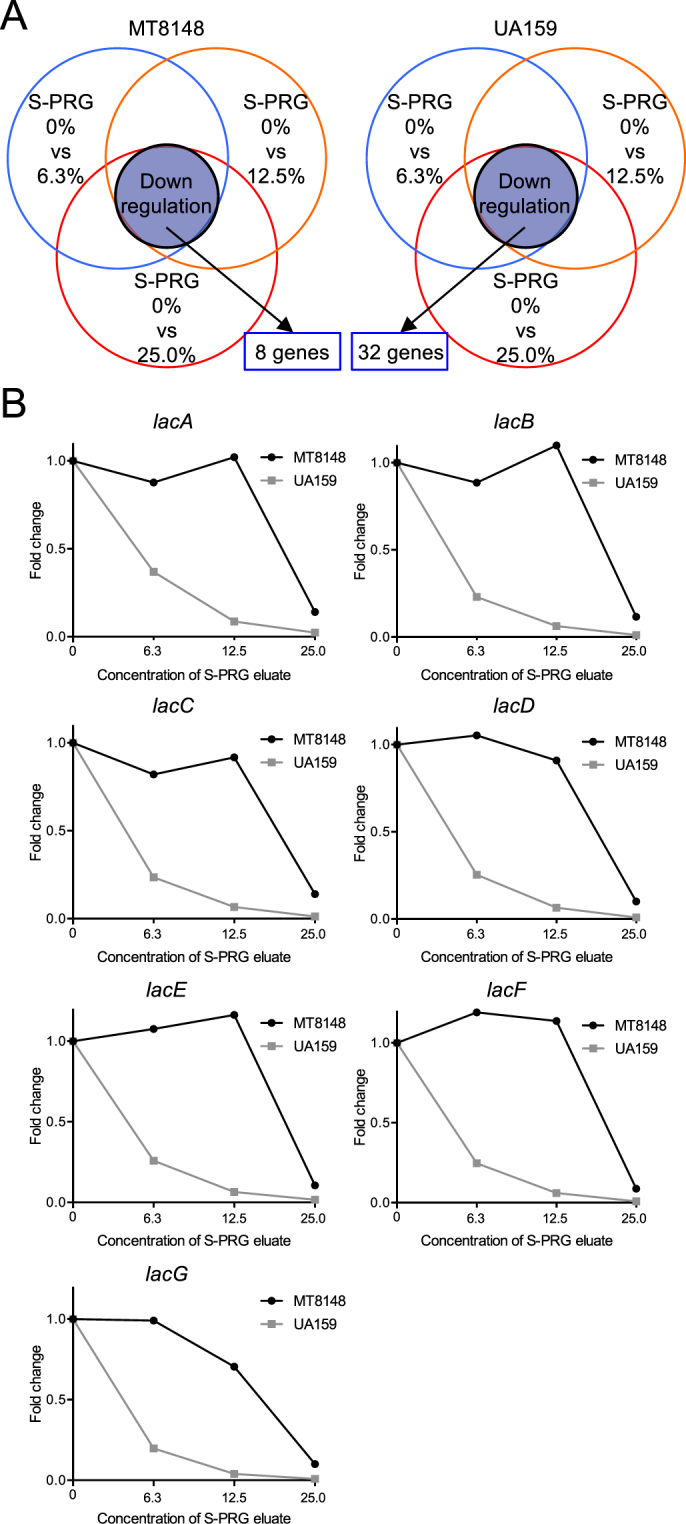
Altered expression of key genes in either *S. mutans* MT8148 or UA159 under three different conditions using DNA microarray analysis. (**A**) Detection of altered genes in the presence of S-PRG eluate. (**B**) Changes in the expression of genes in the *lac* operon of MT8148 and UA159 in the presence of S-PRG eluate.

We also performed DNA microarray analyses under two different conditions focusing on S-PRG eluate concentrations of 0% versus 12.5% and 0% versus 25.0% (Fig. 3A), which revealed that nine genes were downregulated in both MT8148 and UA159 (Tables 3, 4). Among the downregulated genes, four genes encoding the putative glycogen synthase, named *glgA*, *glgB*, *glgC*, and *glg*D^19^, were downregulated by S-PRG eluate in a concentration-dependent manner (Fig. 3B). These genes are involved in glycogen synthesis and the glycogen produced is used for *S. mutans* survival under sugar-starved conditions^19,20^. We further analysed genes prominently regulated under the three conditions at several concentrations of S-PRG eluate: 6.3%, 12.5%, and 25.0%, which were identified in either MT8148 or UA159 (Fig. 4A). Among the 40 genes identified (8 and 32 genes identified in MT8148 and UA159, respectively) (Tables 5, 6), the *lac* operon (*lacA*, *lacB*, *lacC*, *lacD*, *lacE*, *lacF*, *lacG*), which is involved in galactose and lactose metabolism in *S. mutans*^21^, was prominently downregulated in UA159 in a concentration-dependent manner (Fig. 4B). Although the *comY* operon (*comYA*, *comYB*, *comYC*, *comYD*), which is associated with quorum sensing and biofilm formation^22,23^, was downregulated in UA159, the signals and inhibition of the *comY* operon were less prominent when compared to other operons.

**Table 3:** List of downregulated genes of MT8148 in the presence of S-PRG eluate, which were observed in both MT8148 and UA159 under two different conditions using DNA microarray analysis.

Gene	Accession number	S-PRG 0%	S-PRG 6.3%			S-PRG12.5%			S-PRG 25.0%		
		Signal	Signal	Log2*	Change^#^	Signal	Log2*	Change^#^	Signal	Log2*	Change^#^
*celR*	721935.1	34392.8	18718.7	−0.88	0.545	7975.4	−2.11	0.232	11089.6	−1.63	0.323
*glgA*	721880.1	44037.6	25098.8	−0.81	0.570	10246.4	−2.10	0.233	1907.0	−4.53	0.043
*glgB*	721883.1	29635.1	20348.1	−0.54	0.687	6239.4	−2.25	0.211	1451.3	−4.35	0.049
*glgC*	721882.1	45034.7	29677.1	−0.60	0.659	9715.7	−2.21	0.215	1516.4	−4.89	0.034
*glgD*	721881.1	210544.6	147984.5	−0.51	0.703	48733.9	−2.11	0.231	6864.6	−4.94	0.033
*pfl*	720850.1	200380.8	202526.0	0.02	1.011	36209.8	−2.47	0.181	24825.3	−3.01	0.124
*ptcA*	721934.1	21838.2	12188.5	−0.84	0.558	4817.3	−2.18	0.221	6571.4	−1.73	0.301
*ptcC*	721932.1	8470.6	4527.1	−0.90	0.534	2615.5	−1.70	0.309	2737.8	−1.63	0.323
*wapA*	721382.1	3431.3	1740.2	−0.98	0.507	1588.4	−1.11	0.463	1652.3	−1.05	0.482

^*^Log2 means Log2 ratio. ^#^Change means Fold change. The Log2 ratio and Fold change were calculated by signal with each concentration of S-PRG eluate/signal without S-PRG eluate.

**Table 4:** List of downregulated genes of UA159 in the presence of S-PRG eluate, which were observed in both MT8148 and UA159 under two different conditions using DNA microarray analysis.

Gene	Accession number	S-PRG 0%	S-PRG 6.3%			S-PRG12.5%			S-PRG 25.0%		
		Signal	Signal	Log2^*^	Change^#^	Signal	Log2^*^	Change^#^	Signal	Log2^*^	Change^#^
*celR*	721935.1	11936.0	1137.1	−3.39	0.095	366.9	−5.02	0.031	38.4	−8.30	0.003
*glgA*	721880.1	30585.2	17989.6	−0.77	0.588	10161.1	−1.59	0.332	1366.7	−4.48	0.045
*glgB*	721883.1	18468.0	16127.7	−0.20	0.873	8708.1	−1.08	0.471	932.6	−4.31	0.050
*glgC*	721882.1	30765.2	23441.6	−0.39	0.762	10508.8	−1.55	0.342	1055.7	−4.87	0.034
*glgD*	721881.1	134094.9	101989.3	−0.39	0.761	48072.9	−1.48	0.359	5395.6	−4.64	0.040
*pfl*	720850.1	210979.6	166145.4	−0.35	0.787	69347.0	−1.61	0.329	9805.4	−4.43	0.046
*ptcA*	721934.1	7508.6	633.3	−3.57	0.084	207.5	−5.19	0.027	17.7	−8.74	0.002
*ptcC*	721932.1	2921.0	366.2	−3.00	0.125	124.6	−4.56	0.043	38.5	−6.25	0.013
*wapA*	721382.1	3891.4	2323.7	−0.76	0.590	1931.4	−1.01	0.496	568.7	−2.77	0.146

*Log2 means Log2 ratio. ^#^Change means Fold change. The Log2 ratio and Fold change were calculated by signal with each concentration of S-PRG eluate/signal without S-PRG eluate.

**Table 5:** List of downregulated genes of MT8148 in the presence of S-PRG eluate, which were observed in either MT8148 or UA159 under three different conditions using DNA microarray analysis.

Gene	Accession number	S-PRG 0%	S-PRG 6.3%			S-PRG12.5%			S-PRG 25.0%		
		Signal	Signal	Log2*	Change^#^	Signal	Log2*	Change^#^	Signal	Log2*	Change^#^
*bcc*	721410.1	3656.2	2409.2	−0.6	0.659	2386.3	−0.61	0.653	1414.7	−3.81	0.071
*celR*	721935.1	34392.8	18718.7	−0.88	0.545	7975.4	−2.11	0.232	11089.6	−1.63	0.323
*cilA*	721415.1	2407.2	1780.9	−0.43	0.741	2047.7	−0.23	0.851	1084.1	−1.15	0.451
*cilB*	721414.1	2040.5	1377.5	−0.57	0.675	1407.6	−0.53	0.69	719.7	−1.5	0.353
*cilG*	721413.1	1982.2	1594.2	−0.31	0.804	1700.8	−0.22	0.858	719.2	−1.46	0.363
*citC*	721405.1	350.4	534.4	0.61	1.524	757.7	1.11	2.161	165.1	−1.09	0.47
*citG*	721406.1	471.1	806.7	0.78	1.713	1094.5	1.22	2.324	143.2	−1.72	0.303
*citG2*	721416.1	3123.7	2413.7	−0.37	0.772	2832.3	−0.14	0.907	1131.6	−1.47	0.362
*coaA*	721513.1	186.1	250	0.43	1.344	329.3	0.82	1.767	79.9	−1.22	0.429
*comEA*	721053.1	264.2	352.6	0.42	1.335	521.8	0.98	1.975	32.2	−3.04	0.122
*comYA*	722285.1	151.4	221.1	0.55	1.463	343.8	1.19	2.276	22.1	−2.78	0.145
*comYB*	722284.1	253.4	374	0.56	1.479	515.5	1.03	2.042	46.4	−2.44	0.184
*comYC*	722283.1	276.9	452.1	0.71	1.635	684	1.31	2.473	69	−2	0.249
*comYD*	722282.1	147.2	263.5	0.85	1.801	402.7	1.45	2.724	43.7	−1.76	0.295
*galR*	721293.1	1196.1	1062.5	−0.17	0.888	1628.9	0.44	1.36	347.9	−1.78	0.291
*grpE*	720560.1	24097.8	10856.3	−1.15	0.451	10952.4	−1.14	0.454	3039.2	−2.99	0.126
*hsdM*	721299.1	103.1	17.6	−2.55	0.171	14.2	−2.86	0.138	40.4	−1.35	0.392
*lacA*	721844.1	503.8	491.8	−0.04	0.975	691.5	0.46	1.371	77.4	−2.71	0.153
*lacB*	721843.1	1420.2	1345.4	−0.08	0.947	1602.7	0.17	1.129	174.2	−3.03	0.123
*lacC*	721842.1	693.3	681.8	−0.02	0.984	733.5	0.08	1.06	104.1	−2.73	0.15
*lacD*	721841.1	791.7	947.8	0.24	1.184	786.9	−0.01	0.994	88.4	−3.16	0.112
*lacD2*	720591.1	3352.2	1741.7	−0.94	0.52	1563.4	−1.1	0.466	103.9	−5.01	0.031
*lacE*	721839.1	1851.5	2078.7	0.17	1.124	2250.3	0.28	1.216	210.6	−3.14	0.114
*lacF*	721840.1	1250.6	1567.3	0.33	1.253	1506.8	0.27	1.205	127.4	−3.3	0.102
*lacG*	721838.1	7506.5	7801.4	0.06	1.039	5603.4	−0.42	0.747	783.6	−3.26	0.104
*lacX*	721837.1	2089.4	2392.3	0.19	1.143	3630.3	0.8	1.737	211.3	−3.31	0.101
*lytR*	721011.1	2102.5	1188.7	−0.82	0.565	1104.9	−0.93	0.526	507.7	−2.05	0.241
*oadB*	721411.1	4735.5	3705.5	−0.36	0.782	3979.2	−0.25	0.84	2741.2	−0.79	0.578
*pflC*	720930.1	968.8	781.5	−0.31	0.806	904.2	−0.1	0.933	645	−0.59	0.664
*ptcA*	721934.1	21838.2	12188.5	−0.84	0.558	4817.3	−2.18	0.221	6571.4	−1.73	0.301
*ptcB*	721936.1	1549.5	1220.1	−0.35	0.786	1351.8	−0.2	0.872	1410.3	−0.14	0.91
*ptcC*	721932.1	8470.6	4527.1	−0.9	0.534	2615.5	−1.7	0.309	2737.8	−1.63	0.323
*pycB*	721417.1	3315.9	2227.8	−0.57	0.672	2493.8	−0.41	0.752	2372.2	−0.48	0.715
*rgpG*	720710.1	107407.9	3328.3	−5.01	0.031	4296.3	−4.64	0.04	4296.3	−4.64	0.04
*scnE*	722129.1	47	23.3	−1	0.5	14.1	−1.74	0.3	4.2	−3.47	0.09
*spaP*	721042.1	7581.6	3685	−1.04	0.486	1858	−2.03	0.245	1936	−1.97	0.255
*ssb2*	722266.1	673.6	980.5	0.54	1.459	1165.4	0.79	1.733	529	−0.35	0.787
*trk*	721902.1	29059.7	13948.7	−1.06	0.48	6713.2	−2.11	0.231	5386.5	−2.43	0.185
*trkB*	721901.1	21115.7	10376.4	−1.03	0.491	4014.8	−2.4	0.19	3970.1	−2.41	0.188
*wapA*	721382.1	3431.3	1715.5	−1	0.5	1588.4	−1.11	0.463	1652.3	−1.05	0.482

^*^Log2 means Log2 ratio. ^#^Change means Fold change. The Log2 ratio and Fold change were calculated by signal with each concentration of S-PRG eluate/signal without S-PRG eluate.

**Table 6:** List of downregulated genes of UA159 in the presence of S-PRG eluate, which were observed in either MT8148 or UA159 under three different conditions using DNA microarray analysis.

Gene	Accession number	S-PRG 0%	S-PRG 6.3%			S-PRG12.5%			S-PRG 25.0%		
		Signal	Signal	Log2*	Change^#^	Signal	Log2*	Change^#^	Signal	Log2*	Change^#^
*bcc*	721410.1	5980.6	1943.9	−1.62	0.325	1876.8	−1.67	0.314	1108.3	−2.43	0.185
*celR*	721935.1	11936	1137.1	−3.4	0.095	366.9	−5.02	0.031	38.4	−8.3	0.003
*cilA*	721415.1	5421.4	1804.4	−1.59	0.332	1879	−1.53	0.347	726.1	−2.9	0.134
*cilB*	721414.1	3708.8	1459.6	−1.34	0.394	1488.8	−1.32	0.402	712.4	−2.38	0.192
*cilG*	721413.1	4245.9	1734	−1.29	0.408	1999.6	−1.09	0.471	1059.9	−2	0.249
*citC*	721405.1	1927.9	218.7	−3.15	0.113	121.6	−3.99	0.063	164.4	−3.55	0.085
*citG*	721406.1	1543.6	282.1	−2.45	0.183	138.2	−3.48	0.089	183.1	−3.1	0.117
*citG2*	721416.1	5644.4	1906.9	−1.56	0.338	1774.5	−1.67	0.314	694.5	−3.02	0.123
*coaA*	721513.1	126.2	39.1	−1.69	0.31	49.8	−1.34	0.395	54.6	−1.21	0.433
*comEA*	721053.1	135.8	67.9	−1	0.5	29.2	−2.22	0.215	29.5	−2.21	0.217
*comYA*	722285.1	231.2	37.6	−2.65	0.159	17.9	−3.74	0.075	28.8	−3.02	0.124
*comYB*	722284.1	606.2	90	−2.76	0.148	42	−3.87	0.069	69	−3.14	0.113
*comYC*	722283.1	903.7	109.7	−3.05	0.121	45.5	−4.34	0.049	102.2	−3.14	0.113
*comYD*	722282.1	750.9	89.6	−3.13	0.114	38	−4.34	0.05	70.7	−3.43	0.093
*galR*	721293.1	998.6	467	−1.1	0.468	222.2	−2.17	0.222	396.7	−1.33	0.397
*grpE*	720560.1	40392.6	24381	−0.72	0.603	15498.9	−1.38	0.384	23261.7	−0.8	0.576
*hsdM*	721299.1	236.4	230.2	−0.04	0.974	233.2	−0.02	0.986	693.4	1.55	2.933
*lacA*	721844.1	1232.9	487.2	−1.34	0.395	115.2	−3.42	0.093	34	−5.19	0.027
*lacB*	721843.1	3958.7	974.1	−2.02	0.246	263.3	−3.91	0.066	59.5	−6.09	0.015
*lacC*	721842.1	2894.5	707.6	−2.03	0.245	203.3	−3.83	0.07	44.2	−6.07	0.015
*lacD*	721841.1	2529.6	976.9	−1.37	0.386	173.4	−3.88	0.068	25.2	−6.65	0.01
*lacD2*	720591.1	1990.2	422.6	−2.24	0.212	162.4	−3.61	0.082	39.2	−5.67	0.02
*lacE*	721839.1	4352.6	1160.3	−1.91	0.267	303.3	−3.85	0.07	71.2	−5.93	0.016
*lacF*	721840.1	3438.8	936	−1.88	0.272	227.8	−3.92	0.066	38.3	−6.54	0.011
*lacG*	721838.1	30256.7	6224.5	−2.28	0.206	1223.1	−4.63	0.04	270.5	−6.81	0.009
*lacX*	721837.1	2139.1	804.4	−1.41	0.376	251.6	−3.09	0.118	84.2	−4.67	0.039
*lytR*	721011.1	1717.4	772.8	−1.15	0.45	841.5	−1.03	0.49	419.1	−2.04	0.244
*oadB*	721411.1	11315.4	4350.1	−1.38	0.384	4811.2	−1.23	0.425	2660.8	−2.09	0.235
*pflC*	720930.1	1164.2	478.1	−1.28	0.411	391.9	−1.57	0.337	544.7	−1.1	0.468
*ptcA*	721934.1	7508.6	633.3	−3.57	0.084	207.5	−5.19	0.027	17.7	−8.74	0.002
*ptcB*	721936.1	615.6	239.6	−1.37	0.388	103.5	−2.58	0.167	21.7	−4.84	0.035
*ptcC*	721932.1	2921	366.2	−3	0.125	124.6	−4.56	0.043	38.5	−6.25	0.013
*pycB*	721417.1	22423.4	5598.4	−2	0.25	4989.7	−2.17	0.223	2210.2	−3.34	0.099
*rgpG*	720710.1	1621.9	1575.5	−0.04	0.971	1983.1	0.29	1.223	4685.6	1.53	2.889
*scnE*	722129.1	324.3	412.9	0.35	1.273	291.9	−0.15	0.9	284	−0.19	0.876
*spaP*	721042.1	3168.9	3842.9	0.28	1.212	2127.1	−0.58	0.671	2296.4	−0.47	0.724
*ssb2*	722266.1	769.7	369.5	−1.06	0.48	271.3	−1.51	0.352	308.6	−1.32	0.401
*trk*	721902.1	14957.3	11522.8	−0.38	0.77	10018.8	−0.58	0.67	3609	−2.05	0.241
*trkB*	721901.1	10436.8	7856.4	−0.41	0.753	7320	−0.51	0.701	2494.6	−2.06	0.239
*wapA*	721382.1	3891.4	2323.7	−0.74	0.597	1931.4	−1.01	0.496	568.7	−2.78	0.146

^*^Log2 means Log2 ratio. ^#^Change means Fold change. The Log2 ratio and Fold change were calculated by signal with each concentration of S-PRG eluate/signal without S-PRG eluate.

No genes were upregulated in both MT8148 and UA159 under all three eluate conditions tested. DNA microarray analysis employing two different eluate concentrations (Supplemental Fig. 1A) showed only two genes were upregulated with lower fold changes in both MT8148 and UA159 (Supplemental Tables 1 and 2). We further identified six genes in either MT8148 or UA159 under the three different conditions tested (Supplemental Fig. 1B). However, these changes in expressions for all of the genes were relatively small (Supplemental Tables 3 and 4).

### Inhibitory effects of S-PRG eluate in *in vitro* sucrose-dependent adhesion

**Figure 5:**
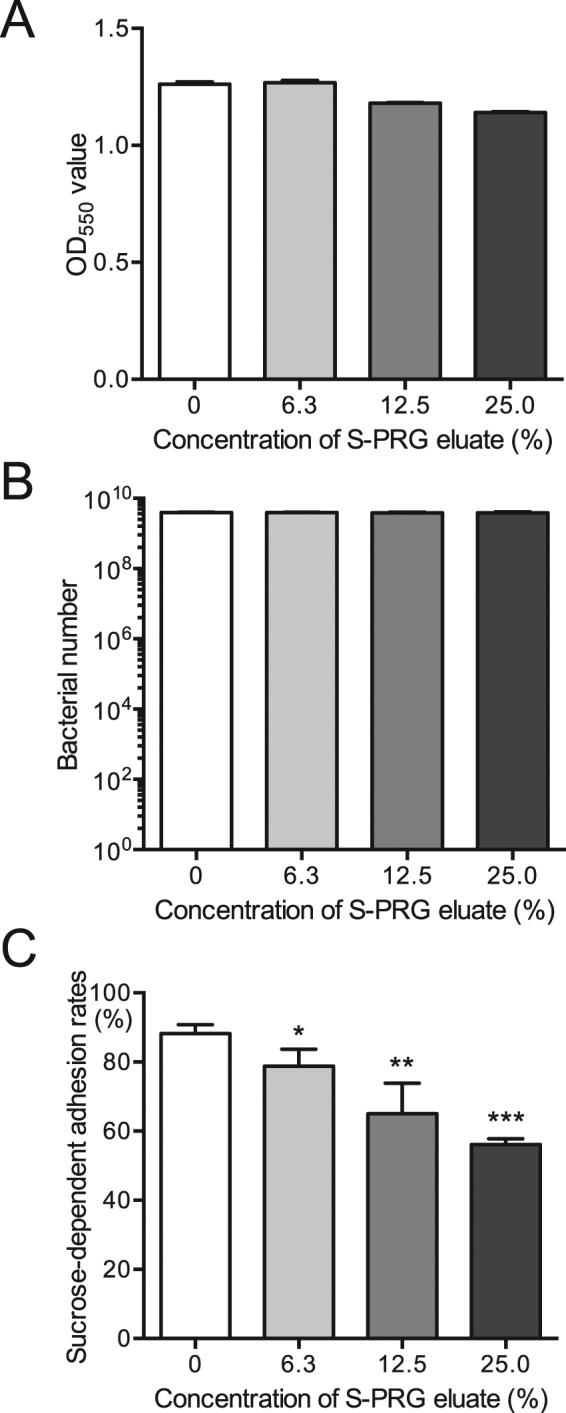
Sucrose-dependent adhesion of *S. mutans* MT8148 in the presence of various concentrations of S-PRG eluate. Bacterial growth for cells used in the sucrose-dependent adhesion assay was determined by OD_550_ values (**A**) and recovered bacterial numbers on MSB plates (**B**). (**C**) Sucrose-dependent adhesion rates. Significant differences were determined using ANOVA with Bonferroni correction. *P < 0.05, **P < 0.01, and ***P < 0.001 versus no S-PRG eluate.

Bacterial suspensions were adjusted in BHI broth containing 1% sucrose to a final concentration of 1.0 × 10^7^ CFU/ml *S. mutans* with or without S-PRG eluate. The bacterial suspensions were then cultured at 37 °C for 18 h at a 30° angle and sucrose-dependent adhesion analysis was performed as previously described^24^. Before the analysis, we confirmed that there were no differences in the total bacterial numbers tested (adhesive cells and non-adhesive cells) in the cultured bacteria among various concentration of S-PRG eluate (Fig. 5A,B). S-PRG eluate significantly inhibited sucrose-dependent adhesion of *S. mutans* in a concentration-dependent manner (*P* < 0.05) (Fig. 5C).

### Inhibitory effects of S-PRG eluate on biofilm formation

**Figure 6:**
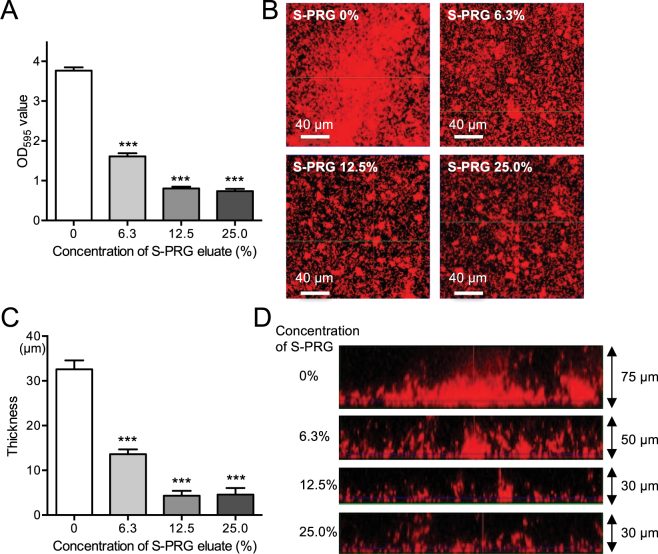
Biofilm formation by *S. mutans* MT8148 grown in BHI with 0.25% sucrose in the presence of various concentrations of S-PRG eluate. (**A**) Quantity of biofilm formation. (**B**) Representative images of formed biofilms using confocal scanning laser microscopy. (**C**) Biofilm thickness. (**D**) Representative images of biofilm thickness using confocal scanning laser microscopy. Significant differences were determined using ANOVA with Bonferroni correction. ***P < 0.001 versus no S-PRG eluate.

Bacterial suspensions were adjusted in BHI broth containing 0%, 0.25% and 1% sucrose to a final concentration of 1.0 × 10^7^ CFU/ml *S. mutans* in the presence or absence of S-PRG eluate. The bacterial suspensions were then added to saliva coated 96-well polystyrene microtiter plates. After incubation at 37 °C for 24 h, biofilms were quantified following staining with crystal violet and the structures of the biofilms were observed by confocal laser scanning microscopy. The quantity of formed biofilms was similar in BHI broth containing 0.25% and 1% sucrose, which were drastically reduced even at a low concentration of S-PRG eluate (Fig. 6A,B, Supplemental Fig. 2A). Both biofilm density and thickness were significantly reduced in the presence of S-PRG eluate, with significant differences (*P* < 0.001) (Fig. 6C,D). On the other hand, only weak biofilms were observed in the absence of sucrose regardless of the presence or absence of S-PRG eluate (Supplemental Fig. 2B).

### Inhibitory effects of S-PRG eluate on cellular hydrophobicity

**Figure 7:**
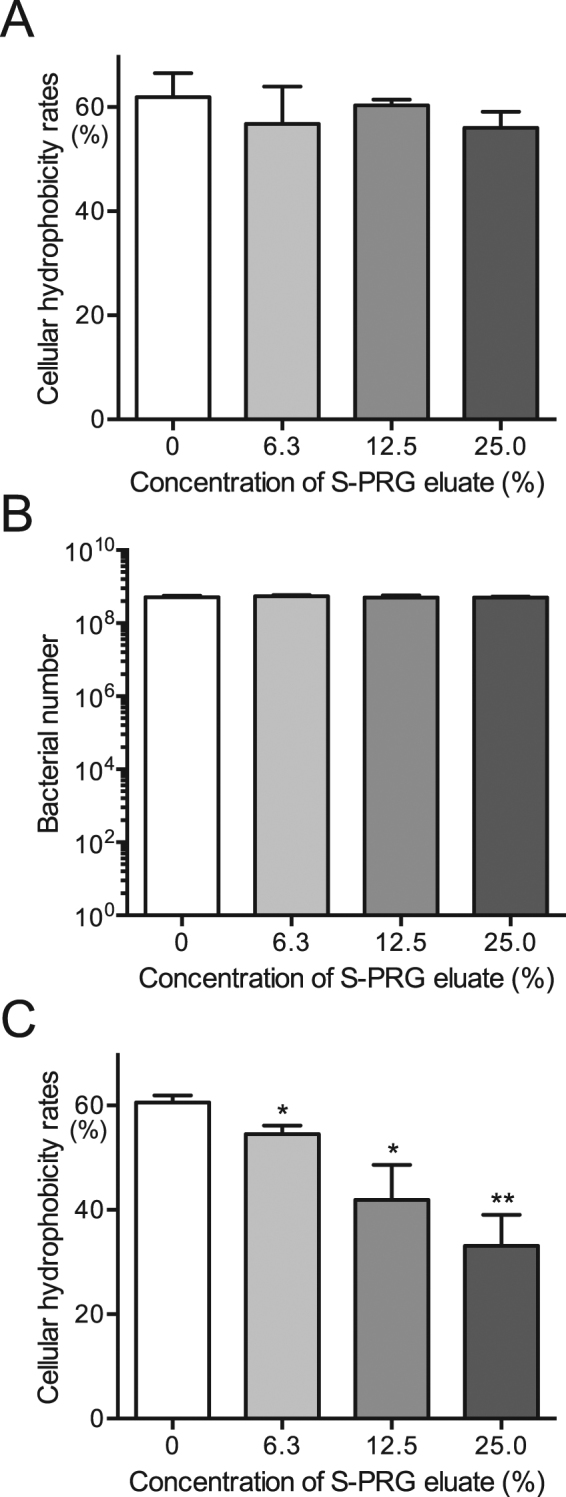
Cellular hydrophobicity of *S. mutans* MT8148 in the presence of various concentrations of S-PRG eluate. (**A**) Cellular hydrophobicity in the presence of S-PRG eluate. (**B**) Bacterial numbers used in the cellular hydrophobicity assay (**C**) Cellular hydrophobicity following the addition of S-PRG eluate 18 h before the assay. Significant differences were determined using ANOVA with Bonferroni correction. *P < 0.05 and **P < 0.01 versus no S-PRG eluate.

Hydrophobic interactions involving *S. mutans* and tooth surfaces play a major role in the sucrose-independent initial adhesion of *S. mutans* to teeth^25^. We determined whether the S-PRG eluate inhibited the hydrophobic properties of *S. mutans* using an *in vitro* cellular hydrophobicity assay, which was assessed by the hydrophobic interaction of *S. mutans* and n-hexadecane^26^. A mixture of each concentration of S-PRG eluate and bacterial cultures adjusted to OD_550_ of 0.6 was vigorously vortexed for 1 min in the presence of n-hexadecane to induce hydrophobic interaction between n-hexadecane and the test strain. Cellular hydrophobicity rates were assessed via n-hexadecane partitioning by *S. mutans* in the presence of various concentrations of S-PRG eluate and showed no significant differences (Fig. 7A). Next, to examine the effects of incubation time on such interactions, we further analysed cellular hydrophobicity using *S. mutans* cells pretreated with S-PRG eluate for 18 h. The S-PRG eluate did not affect bacterial numbers (Fig. 7B), and significantly inhibited cellular hydrophobicity (*P* < 0.05, *P* < 0.01) (Fig. 7C).

### Inhibitory effects of S-PRG eluate against *S. mutans* in post-logarithmic phase

**Figure 8:**
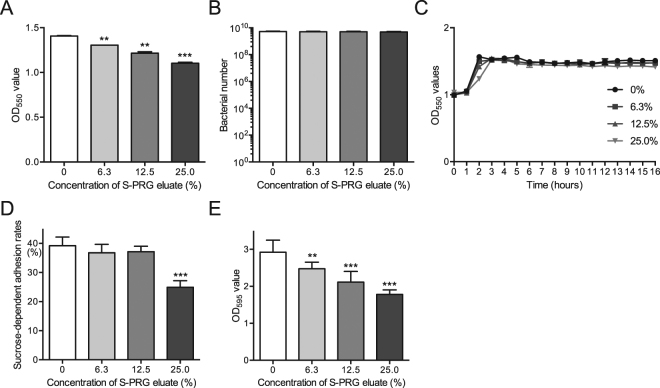
*In vitro* properties of *S. mutans* MT8148 in the late logarithmic phase in the presence of various concentrations of S-PRG eluate. (**A**, **B**) Bacterial growth by adding 1 × 10^9^ CFU/ml of *S. mutans* with 18-h incubation, which was determined by OD_550_ values in BHI broth (**A**) and recovered bacterial numbers on MSB plates (**B**). (**C**) Bacterial growth by adding 1 × 10^9^ CFU/ml of *S. mutans* at multiple time points, which was determined by OD_550_ values in BHI broth. (**D**) Sucrose-dependent adhesion rates. (**E**) Quantitation of biofilm formation. Significant differences were determined using ANOVA with Bonferroni correction. *P < 0.05, **P < 0.01, and ***P < 0.001 versus no S-PRG eluate.

Bacteria in the stationary phase exhibit low sugar metabolic activity^17^. Thus, we investigated the effects of S-PRG eluate on bacterial growth, sucrose-dependent adhesion and biofilm formation using post-logarithmic phase 1.0 × 10^9^ CFU/ml of *S. mutans*. Bacterial numbers were not affected by S-PRG eluate after 18-h incubation although the OD_550_ densities were lower at the higher concentrations of S-PRG eluate in a dose dependent manner (Fig. 8A,B). In addition, bacterial growth in the presence of each S-PRG eluate was monitored and reached the stationary phase within 3-h after incubation (Fig. 8C). Although sucrose-dependent adhesion and biofilm formation by *S. mutans* in the post-logarithmic phase were inhibited in the presence of S-PRG eluate, the inhibitory effects were not as prominent as those using 1.0 × 10^7^ CFU/ml of *S. mutans* prior to the logarithmic growth phase (Fig. 8D,E).

### Inhibitory effects of S-PRG eluate against other streptococci

**Figure 9:**
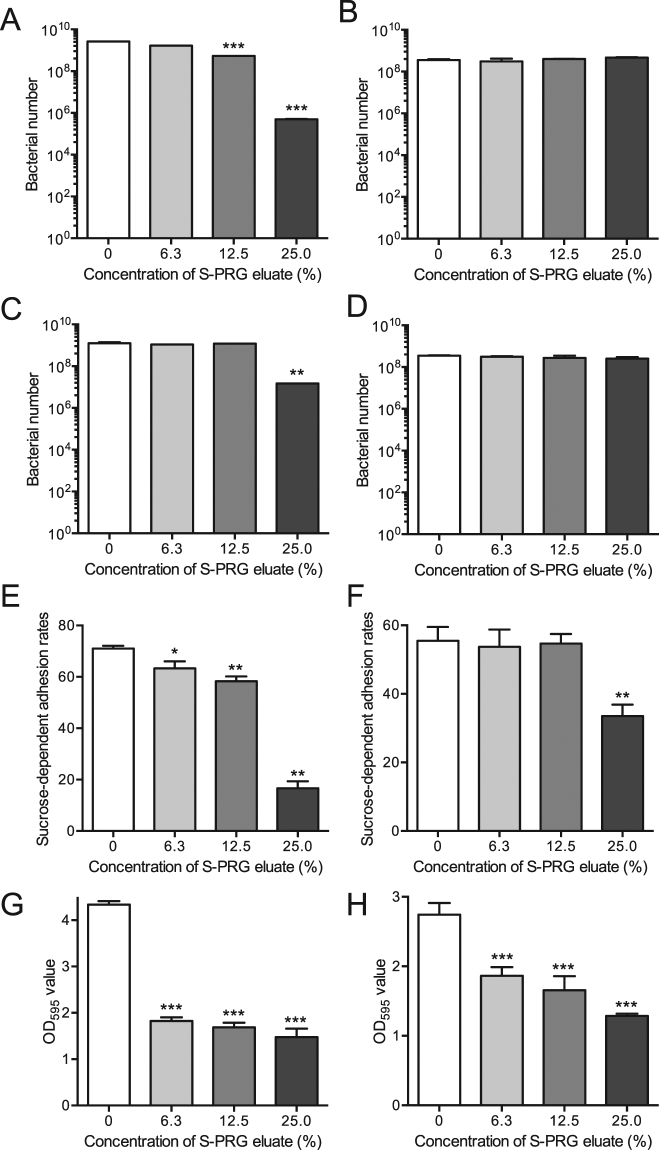
The *in vitro* properties of *Streptococcus sobrinus* B13 and *Streptococcus gordonii* ATCC10558 in the presence of various concentrations of S-PRG eluate. Bacterial growth using *S. sobrinus* before reaching the logarithmic phase (adjusted to 1 × 10^5^ CFU/ml) (**A**) and in the post-logarithmic phase (adjusted to 1 × 10^9^ CFU/ml) (**B**) following 18 h incubation, which was determined by recovered bacterial numbers on MSB plates. Bacterial growth using *S. gordonii* reaching the logarithmic phase (adjusted to 1 × 10^5^ CFU/ml) (**C**) and in the post-logarithmic phase (adjusted to 1 × 10^9^ CFU/ml) (**D**) with 18 h incubations, which was determined by recovered bacterial numbers on MSB plates. Sucrose-dependent adhesion rates using bacterial suspensions of *S. sobrinus* B13 adjusted before reaching the logarithmic phase (adjusted to 1 × 10^7^ CFU/ml) (**E**) and in the post-logarithmic phase (adjusted to 1 × 10^9^ CFU/ml) (**F**), respectively. Quantitation of biofilm formation using bacterial suspensions of *S. sobrinus* B13 adjusted to 1 × 10^7^ CFU/ml (**G**) and 1 × 10^9^ CFU/ml (**H**), respectively. Significant differences were determined using ANOVA with Bonferroni correction. *P < 0.05, **P < 0.01, and ***P < 0.001 versus no S-PRG eluate.

S-PRG eluate prominently repressed the expression of genes encoding the pyruvate dehydrogenase (PDH) complex, and the complex is known to be important for bacterial growth and survival^27^. Thus, we analyzed the inhibitory effect of S-PRG eluate against other oral streptococci using two different types of oral streptococcal species; *Streptococcus sobrinus* B13 which is a *S. mutans*-related species with sucrose-dependent cariogenic properties^28^, and *Streptococcus gordonii* ATCC10558 lacking sucrose-dependent cariogenic properties^29^. *S. sobrinus* before reaching the logarithmic phase (adjusted to 1 × 10^5^ CFU/ml) was inhibited by S-PRG eluate in a concentration-dependent manner (Fig. 9A), whereas inhibition was not observed using the bacteria in the post-logarithmic phase (adjusted to 1 × 10^9^ CFU/ml) (Fig. 9B), similar to what was observed with *S. mutans*. The inhibitory effect on *S. gordonii* in the presence of S-PRG eluate was observed only with the bacterium before reaching the logarithmic growth phase (adjusted to 1 × 10^5^ CFU/ml), though the inhibitory effect was lower than that observed in *S. mutans* and *S. sobrinus* (Fig. 9C,D). In addition, the S-PRG eluate significantly inhibited sucrose-dependent adhesion and biofilm formation by *S. sobrinus* before reaching the logarithmic growth phase (adjusted to 1 × 10^7^ CFU/ml) (*P* < 0.05) (Fig. 9E–H).

## Discussion

Recent studies showed that a S-PRG filler could inhibit the growth and adherence of *S. mutans*^12,13^. However, these studies were performed for the purpose of developing dental products containing S-PRG filler, and not necessarily for antimicrobial activity. In the present study, we analysed the inhibitory effects of S-PRG eluate against *S. mutans*, focusing on the cariogenic properties and virulence genes of the bacterium, which are closely related to dental caries development.

S-PRG filler has been widely used in dental products, mainly contained in sealant and composite resins^7,8^. Although the effects of the presence of each ion component may be different from that in the presence of the other divalent and trivalent ions, releases of all ions from the S-PRG filler in composite resin could be confirmed even after overnight incubation^13^. In addition, the released ions were also identified in whole saliva^12^. Thus, inhibitory effects similar to those of S-PRG eluate observed in the present study may be expected for dental materials containing S-PRG filler, although the exact amounts of the released ions may be different for each clinical situation.

Multiple ions may affect the biological activity for many bacteria^30^. On the other hand, some of the bacteria such as *Psuedomonas aeruginosa* have developed a variety of mechanisms to escape the inhibitory effects of the ions. For example, *P. aeruginosa* can immobilize metal ions outside of the cell, reducing the inhibitory effect of the ions^31^. Conversely, the existence of multiple metal ions sometimes contributes to supporting biofilm formation by bacteria^30,32^. In the present study, a S- PRG eluate could effectively inhibit the growth and *in vitro* cariogenicity of *S. mutans*. In addition, a previous study showed that all of six ions released from a S- PRG filler could inhibit bacterial growth^13^. However, it was reported that some of the proteins of *S. mutans* encodes metal ion transport systems, which are considered as virulence factors for dental caries and infective endocarditis^33,34^. Therefore, further studies should be performed focusing on how the inhibitory effects against *S. mutans* induced by these ions alter bacterial virulence.

One of the most important factors for growth and survival of *S. mutans* is sugar metabolism. The sugar metabolism induced by *S. mutans* occurs through the Embden-Meyerhof-Parnas pathway, followed by acid production from pyruvate^16^. Inhibition of *S. mutans* growth in the presence of S-PRG eluate may result from interference with the Embden-Meyerhof-Parnas pathway or subsequent acid production pathways. To identify the key *S. mutans* genes closely related to the inhibition of growth and survival of the bacterium in the presence of S-PRG eluate, we performed DNA microarray analysis.

Based upon the results of the microarray analysis, S-PRG eluate appeared to strongly downregulate several *S. mutans* genes and the downregulation of some genes in the presence of S-PRG eluate was over 100-fold greater than in the absence of the eluate. Conversely, no gene was upregulated by more than 10-fold in the presence of S-PRG eluate compared with that in the absence of S-PRG eluate. These results indicated that the inhibitory effects on bacterial growth and survival of *S. mutans* in the presence of S-PRG eluate were primarily induced by downregulation of several *S. mutans* genes. Interestingly, three operons (*pdh*, *glg*, and *lac*), all of which play important roles in the survival and sugar metabolism of *S. mutans*, were downregulated in the presence of the S-PRG eluate in a concentration-dependent manner. Thus, these operons may be responsible for the inhibition of bacterial growth and survival observed in the presence of the S-PRG eluate.

Among the genes downregulated by the S-PRG eluate in the DNA microarray analysis, the PDH complex was prominently negatively regulated. The PDH complex converts pyruvate produced following sugar metabolism by *S. mutans* via the Embden-Meyerhof-Parnas pathway to acetyl-CoA and CO_2_^35^. The PDH complex is encoded by an operon consisting of *pdhD*, *pdhA*, *pdhB*, and *pdhC* genes, and a *pdhD* defective mutant strain showed extremely reduced survival as compared with the parent strain^15^. Thus, S-PRG eluate likely affects the *pdhD* gene, resulting in reduced bacterial survival. In addition, the *glg* operon (*glgB*, *glgC*, *glgD*, *glgA*, *glgP*), which is involved in glycogen synthesis from glucose-1-phosphate via ADP-glucose^21,35^, was downregulated in the presence of high concentrations of S-PRG eluate. Although strong inhibition was only observed in UA159, the *lac* operon (*lacA*, *lacB*, *lacC*, *lacD*, *lacF*, *lacE*, *lacG*), which is involved in galactose and lactose metabolic pathways^21^, was also downregulated.

The S-PRG eluate could inhibit several *in vitro* properties related to the development of dental caries in *S. mutans* before the active growth phase. When we further analysed the inhibitory effects of S-PRG eluate against *S. mutans* in the post-logarithmic phase, the effects of the S-PRG eluate were less pronounced. These results indicated that a major role for the S-PRG eluate is the inhibition of *S. mutans* virulence and growth, which occurs before the active growth phase. Therefore, the inhibition of several *in vitro* properties of *S. mutans* before the growth phase in the presence of S-PRG eluate may be result from downregulation of genes such as those in the *pdh* operon and *glg* operons.

Another possible explanation for the inhibitory effects of S-PRG eluate on the *in vitro* cariogenic properties of *S. mutans* is that the S-PRG eluate may affect other *S. mutans* virulence genes since DNA microarray assays were performed under fixed incubation conditions. However, we did test various S-PRG eluate concentrations as well as different *S. mutans* strains. Therefore, further molecular biological analyses should be performed focusing on the inhibitory mechanisms of S-PRG eluate against other relevant *in vitro* cariogenic properties of *S. mutans*.

In the microarray analysis, S-PRG eluate most prominently repressed the expression of genes encoding the pyruvate dehydrogenase (PDH) complex, and the complex is known to be important for the bacterial growth and survival^27^. The PDH complex is also present in various bacterial species such as *Mycobacterium tuberculosis* and *Escherichia coli*^36,37^. Therefore, we thought that other oral streptococcal species may be growth inhibited by the S-PRG eluate prior to their active growth phase rather than following logarithmic growth phase. Based on this hypothesis, we have added the results of bacterial growth with or without S-PRG eluate using *Streptococcus sobrinus* (similar cariogenic bacteria to *S. mutans*) and *Streptococcus gordonii* (non-cariogenic bacteria), both of which were growth inhibited in the presence of S-PRG eluate, especially before the growth phase. On the other hand, some of the cariogenic properties such as sucrose-dependent colonization are specific for *S. mutans* and *S. sobrinus*. Thus, we analyzed the inhibitory effects on several *in-vitro* cariogenic properties of *S. sobrinus* in the presence of S-PRG eluate, which showed that the S-PRG eluate inhibited the cariogenic properties of *S. sobrinus* most prominently prior to active growth phase rather after logarithmic growth similar to that observed with *S. mutans*. These results may indicate that S-PRG eluate can inhibit *S. mutans* as well as various oral streptococci in a similar manner.

In summary, S-PRG eluates inhibited the bacterial growth of *S. mutans* and downregulated genes involved in sugar metabolism. In addition, the S-PRG eluate clearly inhibited several *in vitro* cariogenic properties of *S. mutans*. Taken together, our results suggest that the S-PRG eluate may be a useful tool for reducing dental caries due to its inhibitory effects on the bacterial growth and the sugar-dependent cariogenic properties of *S. mutans*.

## Methods

### S-PRG eluate preparations

S-PRG eluate was prepared as described previously^38^, and was provided by Shofu Inc. (Kyoto, Japan). Briefly, S-PRG filler was mixed with an equal amount of distilled water and mixed gently at room temperature for 24 h, followed by centrifugation at 3,000 × *g*, 23 °C for 6 h to separate the filler and the liquid. The supernatant was then filtered (pore size: 0.45μm) to remove any residual insoluble material and the resulting filtrate was used as the S-PRG eluate. The concentrations of ions released from S-PRG filler except for F^−^ (i.e., Al^3+^, BO_3_^3−^, Na^+^, SiO_3_^2−^, and Sr^2+^) were measured by using an emission spectrophotometer (ICPS-8000, Shimadzu Co., Kyoto, Japan). In addition, the concentration of F^−^ was confirmed with a F^−^ electrode (Model 9609BNWP, Orion Research Inc., Beverly, MA, USA) using an ion selective electrode meter (Model 720 A, Orion Research Inc.). The ion concentrations of the S-PRG eluate were as follows: Al^3+^ = 19.6 ppm, BO_3_^3−^ = 1,656.5 ppm, Na^+^ = 618.5 ppm, SiO_3_^2−^ = 13.9 ppm, Sr^2+^ = 126.8 ppm, and F^−^ = 141.0 ppm. The S-PRG eluate was diluted with brain heart infusion (BHI) broth (Difco Laboratories) at concentration of 25%, 12.5%, 6.3%, and 0%, respectively, and was used in subsequent studies.

### Bacterial strains and growth condition

*S. mutans* strains MT8148 and UA159 were selected from the stock culture collection in our laboratory^39–41^. In addition, *S. sobrinus* B13 and *S. gordonii* ATCC10558 were also used^28,29^. Strains were confirmed to be *S. mutans*, *S. sobrinus* and *S. gordonii* based on their biochemical properties and observation of colony morphologies on MS with and without bacitracin agar plates. These strains were cultured in BHI broth at 37 °C for 18 h and used in subsequent studies.

### Growth inhibition assay

The growth inhibition assays were performed according to methods described previously with some modification^42^. Cells from overnight cultures of *S. mutans* MT8148 grown in BHI broth were collected by centrifugation at 3,000 × *g* for 10 min. We confirmed that bacterial suspensions adjusted in BHI broth to OD_550_ = 1.0 were equal to 1 × 10^9^ CFU/ml by adding serial dilutions of the bacterial suspensions onto MSB agar plates. Using the suspensions, bacterial dilutions with final concentrations ranging from 1.0 × 10^3^ to 1.0 × 10^8^ CFU/ml with or without S-PRG eluate were prepared. After 18-h incubation at 37 °C, bacterial growth was measured at OD_550_ and bacterial suspensions were then streaked onto MSB plates supplemented with 15% (w/v) sucrose and 0.2 U/ml of bacitracin and incubated anaerobically at 37 °C for 48 h. The numbers of colonies were counted after identifying the characteristic colony morphology of mutans streptococci. As for *S. sobrinus* B13 and *S. gordonii* ATCC10558, the inhibitory effects of S-PRG eluate on bacterial suspensions with concentrations of 1.0 × 10^5^ CFU/ml were also analysed, since *S. mutans* was significantly inhibited by S-PRG eluate at this density. In addition, to monitor the kinetics of bacterial growth for MT8148, OD_550_ values of the bacterial suspensions were adjusted 1.0 × 10^7^ CFU/ml with or without S-PRG eluate and monitored every 1 hour until the bacterial growth reached the stationary phase. In addition, survival of 1.0 × 10^7^ CFU/ml of the bacteria added to BHI in the presence of each concentration of S-PRG eluate was monitored for 2 weeks by adding aliquots of the bacterial suspensions to MSB plates every 48 hours.

The growth of *S. mutans* MT8148 in late logarithmic phase was determined by a previously described method^43^. *S. mutans* MT8148, which was grown for 24 h to reach stationary phase, was adjusted to a final concentration of 1.0 × 10^9^ CFU/ml in BHI broth with or without S-PRG eluate. After a further 18-h incubation at 37 °C, bacterial growth was measured at OD_550_ and bacterial suspensions were streaked onto MSB plates, followed by colony counting as described above. In addition, to monitor the kinetics of bacterial growth, the bacterial suspensions adjusted to a final concentration of 1.0 × 10^9^ CFU/ml with or without S-PRG eluate were cultured at 37 °C and OD_550_ values of the bacterial suspensions were measured every hour until the bacteria entered the stationary phase. As for *S. sobrinus* B13 and *S. gordonii* ATCC10558, 1.0 × 10^9^ CFU/ml of the bacteria were incubated with S-PRG eluate at 37 °C for 18 h and the bacterial suspensions were then streaked onto MSB plates, followed by colony counting as described above.

### DNA microarray assays

A systematic analysis of gene alterations in *S. mutans* was performed using DNA microarrays to identify *S. mutans* gene expression affected by the S-PRG eluate, as described previously^44^. Briefly, 1.0 × 10^7^ CFU/ml of *S. mutans* MT8148 and UA159 in BHI broth were cultured with each specified concentration of S-PRG eluate at 37 °C for 18 h. Amino-allyl amplified RNA was then obtained from total RNA using the Amino-allyl MessageAmp aRNA kit (Ambion, Inc., Austin, TX, USA). The purity, concentration, and quality of the RNA samples were confirmed with a NanoDrop One (Thermo Fisher Scientific) and Agilent 2100 bioanalyser (Agilent Technologies, Inc., Santa Clara, CA, USA). The purity and quality of RNA were assessed by nucleic acid absorbance at A_260_/A_230_ and A_260_/A_280_ of each sample and were more than 2.0, respectively. The concentrations of RNA of these samples ranged from 50 to 440 ng/μl. All samples were used for microarray assays after adjustment to a minimum concentration (50 ng/μl). The microarray assays were carried out by Takara Bio. Inc. (Otsu, Japan) using products for microarray analysis manufactured by Agilent Technologies, according to the manufacturer’s protocols. Briefly, Cy3 complimentary RNA was labelled with a Low Input Quick Amp Labeling Kit, One-Color (Agilent Technologies), and the Cy3-Labeled complimentary RNA was hybridized with the complete genome of *S. mutans* UA159 assembled with the Agilent Expression Array kit. After washing with Gene Expression Wash Buffers Pack (Agilent Technologies), the hybridization images were analysed using an Agilent Microarray Scanner (G2565CA) (Agilent Technologies). Quantitative data were obtained using Agilent Feature Extraction software (Agilent Technologies) and corrections for background signal intensity for the data were performed using a method, previously described^45^. Altered genes were identified using three different comparisons for each *S. mutans* strain and focused on the following S-PRG eluate concentrations: 0% versus 6.3%, 0% versus 12.5%, and 0% versus 25.0%. In addition, altered genes using two different comparisons for each *S. mutans* strain were also determined using S-PRG eluate concentrations of 0% versus 12.5% and 0% versus 25.0%. We selected genes with changes of increase or decrease greater than 1.0 of Log2 ratio in presence or absence of the indicated concentrations of eluate.

### Sucrose-dependent adhesion

Sucrose-dependent adhesion to a glass surface was analysed as previously described with some modification^24^. Cells from overnight cultures of *S. mutans* MT8148 and *S. sobrinus* B13 were collected by centrifugation at 3,000 × *g* for 10 min, respectively. Cultures were adjusted in BHI broth containing 1% sucrose to a final concentration of 1.0 × 10^7^ CFU/ml with or without S-PRG eluate. Bacterial suspensions were then cultured at 37 °C for 18 h at a 30° angle. After incubation, both adhesive and non-adhesive cells were measured by OD_550_ values and were streaked onto MSB plates to confirm the bacterial number in the culture tubes. In addition, culture tubes were also prepared other than those for the confirmation of the bacterial numbers were used for the sucrose-dependent adhesion assay. The culture tubes were vigorously vibrated with a vortex mixer for 3 s and non-adhesive cells were transferred to fresh tubes. Cells remaining on the glass surface (adhesive cells) were removed using a rubber scraper and suspended in 3 ml of water. Both adhesive and non-adhesive cells were dispersed by ultrasonication, and the cell masses were determined by densitometry at OD_550_. Total cells were calculated as OD_550_ (adhesive cells + non-adhesive cells), and the percent adherence was calculated as 100 × OD_550_ (adhesive cells)/OD_550_ (total cells).

Sucrose-dependent adhesion of *S. mutans* MT8148 and *S. sobrinus* B13 in the late logarithmic phase was determined according to a method described previously^43^. These bacteria were grown for 24 h to reach the stationary phase and were adjusted to a final concentration of 1.0 × 10^9^ CFU/ml in BHI broth containing 1% sucrose with or without S-PRG eluate. Aliquots were cultured at 37 °C for 18 h at a 30° angle. Adherence rates were calculated as described above.

### Biofilm assay

The quantity of formed biofilms was assessed as previously described with some modification^46,47^. Human saliva collected from a healthy volunteer was centrifuged at 12,000 × *g* for 10 min and the supernatant was filtered (pore size: 0.45 μm). The supernatant was diluted 1:3 with Milli-Q water to produce 25% saliva and coated 96-well polystyrene microtiter plates for 2 h. Cells from overnight cultures of *S. mutans* MT8148 or *S. sobrinus* B13 were collected by centrifugation at 3,000 × *g* for 10 min. Cultures were adjusted to 1.0 × 10^7^ CFU/ml in BHI broth containing 0%, 0.25% and 1% sucrose with or without S-PRG eluate. Next, 200 µl of the bacterial suspensions were added to 96-well polystyrene microtiter plates coated with human saliva. After incubation at 37 °C for 24 h, the plates were washed three times with phosphate-buffered saline (PBS) to remove loosely bound bacteria. Biofilms were fixed with 25% formaldehyde for 10 min and stained with 1% crystal violet in water (Sigma-Aldrich) for 15 min at room temperature. Next, the plates were washed three times and dissolved in 95% ethanol before quantification of the absorbance at 595 nm with an enzyme-linked immunosorbent assay microplate reader (Thermo Fisher Scientific, Waltham, MA, USA).

Biofilm formation by *S. mutans* MT8148 and *S. sobrinus* B13 in late logarithmic phase was determined as follows. The bacteria were grown for 24 h to reach the stationary phase and adjusted in BHI broth containing 0.25% sucrose to a final concentration of 1.0 × 10^9^ CFU/ml with or without S-PRG eluate. Next, 200 µl of the bacterial suspensions were added to 96-well plates coated with 25% saliva followed by incubation at 37 °C for 24 h. Analysis of biofilm formation was then performed as described above.

### Microscopic observation of *in vitro* biofilms

Quantitative and structural analysis of biofilms by confocal laser scanning microscopy was performed as described previously with some modifications^48^. Cells from overnight cultures of *S. mutans* MT8148 were collected by centrifugation at 3,000 × *g* for 10 min. Bacterial cells were next resuspended in 1 ml of Milli-Q water with 5 µl of 10 mM hexidium iodide (Invitrogen, Carlsbad, CA, USA) and incubated in the dark for 15 min at room temperature. The bacterial suspension was adjusted in BHI broth containing 0.25% sucrose to each specified cell concentration with or without S-PRG eluate. Next, 200 µl of the bacterial suspension were added to a chambered cover glass system (CultureWell^TM^, Grace Bio Labs, Bend, OR, USA) coated with filtered human saliva. The chamber was then incubated at 37 °C for 18 h in the dark. At the end of the experimental period, non-attached *S. mutans* cells were washed with PBS and biofilms were observed by confocal scanning laser microscopy using a TCS-SP5 microscope (Leica Microsystems GmbH, Wetzlar, Germany) with reflected laser light at 488 nm, as well as a DMI6000 B fluorescence microscope (Leica) and a 63× oil immersion objective. Biofilm thickness was measured as follows: an image taken in the z-axis was divided into 10 sections and the thickness of the central portion was measured. The mean value and standard deviation for the thickness were then calculated.

### Hydrophobic interaction

Cellular hydrophobicity was determined using n-hexadecane (Wako) as previously described with some modifications^26^. Overnight cultures of *S. mutans* MT8148 were collected by centrifugation at 3,000 × *g* for 10 min. Cultures were adjusted to OD_550_ of 0.6 with or without S-PRG eluate Next, 0.2 ml of n-hexadecane was added to 3 ml of bacterial cells and then uniformly agitated with a vortex mixer for 1 min to induce hydrophobic interaction between the test strain and n-hexadecane. The mixture was left to stand for 10 min at room temperature and the optical density of the aqueous phase was determined at OD_550_. The incorporation rate was calculated as follows: [1 − OD_550_ (aqueous phase of the tube containing the cell suspensions with added n-hexadecane)/OD_550_ (aqueous phase of the tube containing only cell suspensions)] × 100 (%)]. The results are shown as the mean ± SD from four independent experiments.

Cellular hydrophobicity using pre-logarithmic *S. mutans* cells pretreated with S-PRG eluate was also evaluated as follows. *S. mutans* MT8148 was added at a final concentration of 1.0 × 10^7^ CFU/ml in BHI broth with or without S-PRG eluate. After the bacterial suspensions were incubated at 37 °C for 18 h, cultures were adjusted to OD_550_ of 0.6 and the bacterial suspensions were streaked onto MSB plates to confirm the bacterial numbers. Additional bacterial cultures adjusted to OD_550_ of 0.6 with or without S-PRG eluate were prepared to use for the following the cellular hydrophobicity assays, as described above.

### Statistical analysis

Statistical analyses were conducted using GraphPad Prism 6 (GraphPad Software Inc., La Jolla, CA, USA). Intergroup differences were analysed using an analysis of variance (ANOVA) with Bonferroni correction. Results were considered to be significantly different at *P* < 0.05.

## Electronic supplementary material

Supplementary Information
