# Supplementary material for: Inhibitory effect of surface pre-reacted glass-ionomer (S-PRG) eluate against adhesion and colonization by Streptococcus mutans
Source: Sci Rep. 2018 Mar 22;8:5056. doi: 10.1038/s41598-018-23354-x (PMC5864963; doi:10.1038/s41598-018-23354-x)
Supplement: Supplementary file 1 — Supplementary Information [file 41598_2018_23354_MOESM1_ESM.pdf]

# **Inhibitory effect of surface pre-reacted glass-ionomer (S-PRG) eluate against adhesion and colonization by *Streptococcus mutans***

Ryota Nomura*, Yumiko Morita, Saaya Matayoshi, and Kazuhiko Nakano

**Supplementary Table 1. List of upregulated genes of MT8148 in the presence of S-PRG eluate, which were observed in both MT8148 and UA159 under two different conditions using DNA microarray analysis.**

| gene | accession number | S-PRG 0% | S-PRG 6.3% | | | S-PRG12.5% | | | S-PRG 25.0% | | |
|---|---|---|---|---|---|---|---|---|---|---|---|
| | | signal | signal | Log2* | change# | signal | Log2* | change# | signal | Log2* | change# |
| *citZ* | 721099.1 | 2011.8 | 9341.3 | 2.22 | 4.649 | 10155.8 | 2.34 | 5.052 | 11623.9 | 2.53 | 5.784 |
| *ylxM* | 721453.1 | 256.0 | 466.9 | 0.89 | 1.851 | 848.2 | 1.75 | 3.359 | 961.5 | 1.93 | 3.809 |

*Log2 means Log2 ratio. #Change means Fold change. The Log2 ratio and Fold change were calculated by signal with each concentration of S-PRG eluate/ signal without S-PRG eluate.

**Supplementary Table 2. List of upregulated genes of UA159 in the presence of S-PRG eluate, which were observed in both MT8148 and UA159 under two different conditions using DNA microarray analysis.**

| gene | accession number | S-PRG 0% | S-PRG 6.3% | | | S-PRG12.5% | | | S-PRG 25.0% | | |
|---|---|---|---|---|---|---|---|---|---|---|---|
| | | signal | signal | Log2* | change# | signal | Log2* | change# | signal | Log2* | change# |
| *citZ* | 721099.1 | 2981.0 | 3850.2 | 0.37 | 1.292 | 7838.3 | 1.40 | 2.630 | 7871.8 | 1.40 | 2.642 |
| *ylxM* | 721453.1 | 323.5 | 563.3 | 0.80 | 1.735 | 652.0 | 1.01 | 2.016 | 1073.9 | 1.73 | 3.322 |

*Log2 means Log2 ratio. #Change means Fold change. The Log2 ratio and Fold change were calculated by signal with each concentration of S-PRG eluate/ signal without S-PRG eluate.

**Supplementary Table 3. List of upregulated genes of MT8148 in the presence of S-PRG eluate, which were observed in either MT8148 or UA159 under three different conditions using DNA microarray analysis.**

| gene | accession number | S-PRG 0% | S-PRG 6.3% | | | S-PRG12.5% | | | S-PRG 25.0% | | |
|---|---|---|---|---|---|---|---|---|---|---|---|
| | | signal | signal | Log2* | change# | signal | Log2* | change# | signal | Log2* | change# |
| *citZ* | 721099.1 | 2011.8 | 9341.3 | 2.22 | 4.649 | 10155.8 | 2.34 | 5.052 | 11623.9 | 2.53 | 5.784 |
| *glnA* | 720816.1 | 3913.3 | 8827.4 | 1.18 | 2.258 | 10800.8 | 1.47 | 2.764 | 10028.9 | 1.36 | 2.566 |
| *idh* | 721100.1 | 1161.3 | 3778.6 | 1.70 | 3.252 | 4400.5 | 1.92 | 3.791 | 2413.4 | 1.06 | 2.079 |
| *ilvH* | 720697.1 | 2135.5 | 2568.7 | 0.27 | 1.204 | 3170.3 | 0.57 | 1.486 | 11440.8 | 2.42 | 5.364 |
| *purD* | 720531.1 | 1873.4 | 2540.2 | 0.44 | 1.359 | 4315.1 | 1.21 | 2.309 | 2061.7 | 0.14 | 1.102 |
| *purK* | 720534.1 | 5543.8 | 9380.9 | 0.76 | 1.693 | 16232.1 | 1.55 | 2.929 | 7709.8 | 0.48 | 1.391 |

*Log2 means Log2 ratio. #Change means Fold change. The Log2 ratio and Fold change were calculated by signal with each concentration of S-PRG eluate/ signal without S-PRG eluate.

**Supplementary Table 4. List of upregulated genes of UA159 in the presence of S-PRG eluate, which were observed in either MT8148 or UA159 under three different conditions using DNA microarray analysis.**

| gene | accession number | S-PRG 0% | S-PRG 6.3% | | | S-PRG12.5% | | | S-PRG 25.0% | | |
|---|---|---|---|---|---|---|---|---|---|---|---|
| | | signal | signal | Log2* | change# | signal | Log2* | change# | signal | Log2* | change# |
| *citZ* | 721099.1 | 2981.0 | 3850.2 | 0.37 | 1.292 | 7838.3 | 1.40 | 2.630 | 7871.8 | 1.40 | 2.642 |
| *glnA* | 720816.1 | 4433.9 | 4162.9 | -0.09 | 0.939 | 15224.5 | 1.78 | 3.437 | 4554.6 | 0.04 | 1.027 |
| *idh* | 721100.1 | 1210.9 | 1786.2 | 0.56 | 1.475 | 2576.9 | 1.09 | 2.128 | 1676.8 | 0.47 | 1.385 |
| *ilvH* | 720697.1 | 4203.5 | 9367.9 | 1.16 | 2.231 | 8865.0 | 1.08 | 2.112 | 9574.6 | 1.19 | 2.280 |
| *purD* | 720531.1 | 955.3 | 2130.0 | 1.16 | 2.231 | 3688.3 | 1.95 | 3.863 | 2024.7 | 1.08 | 2.120 |
| *purK* | 720534.1 | 2146.5 | 4975.6 | 1.21 | 2.319 | 8335.2 | 1.96 | 3.886 | 5267.3 | 1.30 | 2.455 |

*Log2 means Log2 ratio. #Change means Fold change. The Log2 ratio and Fold change were calculated by signal with each concentration of S-PRG eluate/ signal without S-PRG eluate.

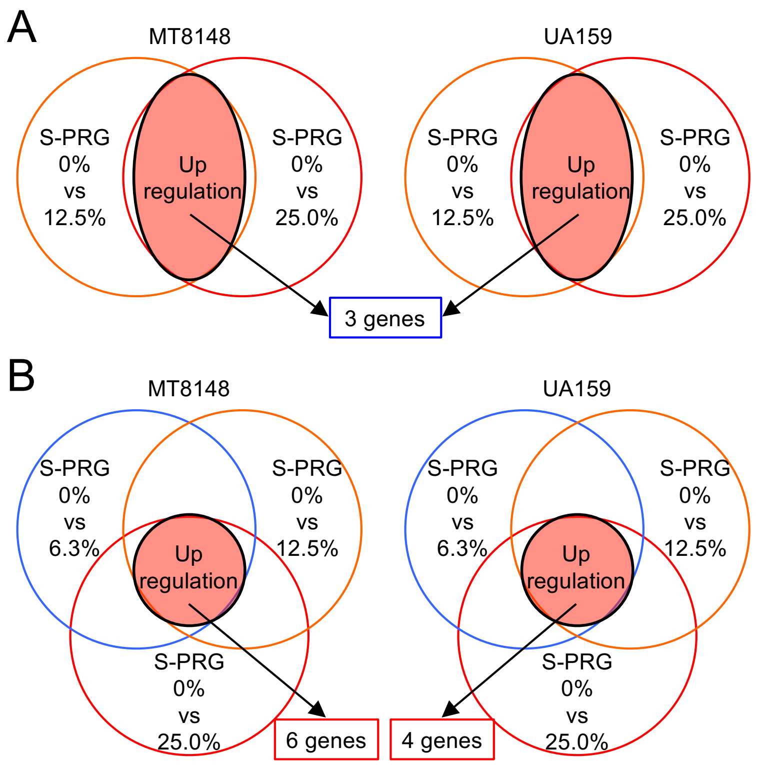


**Supplemental Figure 1.** Selection of altered genes in the presence of S-PRG eluate using DNA microarray analysis. (A) Altered expression of key genes in both *S. mutans* MT8148 and UA159 under two different conditions. (B) Altered expression of key genes in either *S. mutans* MT8148 or UA159 under three different conditions.

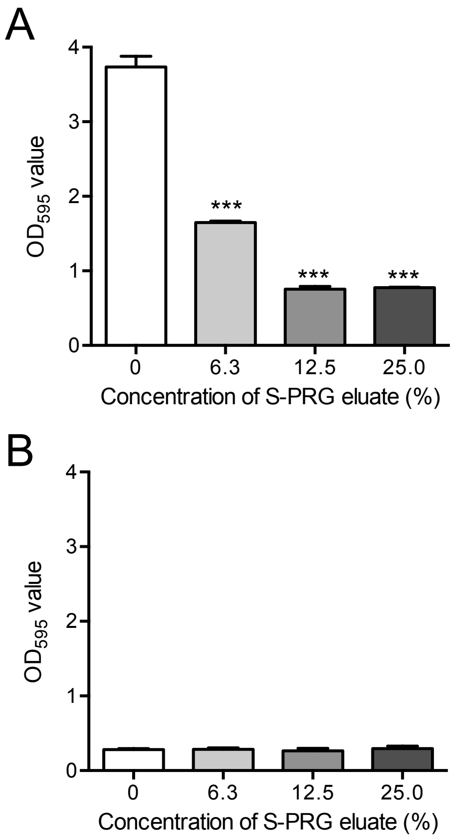


**Supplemental Figure 2.** Quantitation of biofilm formation by *S. mutans* MT8148 in the presence of various concentrations of S-PRG eluate with or without 1% sucrose. Biofilm formation by cells grown in BHI in the presence of 1% sucrose (A) and without sucrose (B).
